# The challenges of climate change and human impacts faced by Mexican coasts: A comprehensive evaluation

**DOI:** 10.1371/journal.pone.0320087

**Published:** 2025-04-17

**Authors:** María Luisa Martínez, Rodolfo Silva, Valeria Chávez, Jorge López-Portillo, Karla Salgado, Etzaguery Marín-Coria, Octavio Pérez-Maqueo, Carmelo Maximiliano-Cordova, Rosario Landgrave, Víctor de la Cruz

**Affiliations:** 1 Instituto de Ingeniería, UNAM, Ciudad de México, Mexico; 2 Instituto de Ecología, A.C., Xalapa, Mexico; Institute of Oceanology Chinese Academy of Sciences, CHINA

## Abstract

The extensive shoreline of Mexico is heterogeneous and diverse, but it is increasingly exposed to degradation and loss. This is the first study performed at a national level and with a multidisciplinary approach, that aims to assess the impact of climate change and human-related pressures affecting Mexican coasts. From 1863 to 2022, 386 tropical cyclones have landed on Mexican coasts, six of them of category 5 (most on the Atlantic). Sea level rise projections showed that the Atlantic coast is the most vulnerable, whereas intense coastal erosion ( > 25m/year) is more widespread on the northern Pacific coast. Human impacts include coastal urbanization, ecosystem degradation and coastal armouring. Six million people live on Mexican coasts, mostly in the Caribbean. Mangroves and coastal dunes each cover nearly 800,000 ha. The mangroves are relatively well preserved, but almost half the area of the coastal dunes is degraded. Coastal armouring is widespread along the coasts, but most of these structures (55%) are found on the Yucatan peninsula. Activities required to improve the condition of Mexican coasts and make them a sustainable place to live would include: adaptation of human settlements to the conditions of the dynamic coasts; appropriate coastal protection measures that do not induce downdrift erosion; dealing with coastal risks by restoring and preserving coastal ecosystems.

## 1. Introduction

Recent global analyses reveal large-scale degradation of many of the coastal ecosystems that are valuable for flood defense [1,2], while the economic value and population density in Low Elevation Coastal Zones (i.e., regions up to 10 meters above sea level) continue to increase [3], often putting further pressure on these systems. There is therefore a need to fundamentally rethink our coastal management strategies at local and national level. For muddy coasts, the use of mangroves and salt marshes for flood defense, has been extensively reviewed [4]. However, for sandy coasts, innovations, such as mega nourishments [5], or using seagrasses to prevent erosion [6], have been implemented in individual studies only; a broader perspective is lacking.

In this sense, the biophysical state of beach-dune ecosystems can be considered as an indicator of the health of the surrounding ecosystems as they function as an ecological membrane [7,8]. The diagnosis of mass and energy fluxes on the coasts is essential for their optimal management [9]. Monitoring is fundamental in this process [10]. The combination of local (e.g., coastal defences) and regional impacts (e.g., dam construction), along with uncertainties about rates of sea level rise and climate change accentuate the need for affordable future flood defenses [10,11]. For the development of appropriate legislation and the use of governance arrangements that facilitate adaptation to changing needs, particularly in developing countries, e.g., [12], inexpensive national data is needed.

Mexico is privileged to have coastlines fronting two oceans: the Pacific in the west and the Atlantic in the east, amounting to over 11,000 km, third in the Americas [13,14]. Along Mexico’s coasts there are a wide range of ecosystems: coral reefs, seagrass beds, mangroves, sandy beaches, and coastal dunes, all contributing to the high biodiversity found in Mexico. These ecosystems provide unique services, such as the filtration of large volumes of seawater [15], storm protection [4,16], recreation and scenic beauty [17,18], nutrient cycling [19], the filtration of pollutants [7], sand production [6,20], the support of coastal fisheries [21] and provision of critical habitats for many endangered species, including birds (such as flamingoes), turtles and some invertebrates (such as the horseshoe crab) [22].

As in many countries, a wide range of climate change- and human-related pressures affect the coasts of Mexico. Increasingly, these pressures cause extreme conditions that impact natural ecosystems, human settlements, and infrastructure. Hence, the recovery of the coasts and their ecosystems is increasingly complex, and the likelihood of reaching a point of no return becomes more probable as environmental degradation grows.

### 1.1. Climate change and environmental dynamics

#### 1.1.1. Tropical cyclones.

These naturally drive the dynamics of the coasts and their ecosystems. The energetic waves, storm surges, and strong winds shape and modify the coastal geomorphology. However, when they affect human lives and assets, these are seen as hazards and natural catastrophes. Mexico is one of the countries most impacted by tropical cyclones, which land on both shores [23] every year. Since 1902, they have caused 4,477 casualties and $ 27,178,977 (USD x 1,000) in economic damages [24]. Storm-related risks are relatively high in Mexico since over 10 million people live less than 10 km from the shoreline [25], with a high incidence of tropical storms. Recent climate-change related trends indicate that tropical cyclones are becoming more frequent and intense, thus increasingly affecting the coasts.

#### 1.1.2. Sea level rise (SLR).

Sea level is a sensitive index of climate change and variability. A previous study on Mexico’s Atlantic coasts [26] showed that 30% of tourist destinations are exposed to flooding induced by climate change-related events such as sea level rise. Furthermore, 66% of the hotels on these coasts are on squeezed beaches, pointing to a potential socioeconomic problem looming for Mexico’s sun, sand, and sea tourist destinations.

#### 1.1.3. Shoreline change.

The interaction between the sand, tides, waves, and wind means that erosion and accretion naturally occur on the coasts. These natural and episodic phenomena are generally associated with erosion, a natural coastal process. However, the sand returns to the coast during calm conditions. Consequently, the beach and dunes will recover if the sediment budget is unaffected by natural or human-related processes [27]. Nevertheless, the combination of sea level rise and increased storminess, with human-induced alterations of coastal sediment supply and transport processes, tend to increase shoreline erosion, with the coasts generally migrating inland [28]. Although erosion is a natural process on the coast, it becomes problematic for coastal settlements and infrastructure.

### 1.2. Human impact

#### 1.2.1. Coastal populations and settlements.

Coastal populations and settlements [29] analyzed urban settlement patterns in the Low Elevation Coastal Zone (LECZ), the contiguous area along the coast less than 10 meters above sea level. These authors report that this zone covers 2 percent of the world’s land area but contains 10 percent of the world’s population and 13 percent of the world’s urban population. Mexico is no exception to this pattern. Despite the ecological importance of its coasts, inadequate management has promoted human encroachment along the shores. The accelerated expansion of sun, sand, sea tourism in the last 50 years has increased the coasts’ socioeconomic significance, mainly on sandy beaches [30]. The continuous and mostly disordered urban sprawl on some coastlines has resulted in habitat degradation or loss and terrestrial and oceanic pollution. Human encroachment is exposing the coasts to pressures at unprecedented scale and intensity [29,31].

#### 1.2.2. Coastal armoring.

Ideally, the conflict between natural coastal dynamics and human assets would result in the retreat inland of human settlements and infrastructure to allow the dynamic natural functioning of coastal ecosystems, providing storm protection. However, for socioeconomic reasons, this retreat is generally impossible. Consequently, other actions are implemented, such as beach nourishment and coastal armoring (building infrastructure for coastal protection) [32]. These human modifications limit the flexibility and functionality that coastal ecosystems need to respond to the naturally extreme conditions of the coast [28]. In addition, the ecological consequences of inadequate engineering actions can exacerbate downdrift erosion and induce biodiversity loss.

In brief, we can say that Mexico’s coasts are facing pressures from different origins that expose them to increasingly extreme conditions. Detailed studies have yet to analyze these pressures at a national level to assess the status of the coasts, as well as the knowledge and gaps in mitigation and management practices across different coastal ecosystems. Given this background, the goals of this work were threefold. First, we aimed to analyze the environmental dynamics of the Mexican coasts, considering climate change-related impacts such as the occurrence and frequency of tropical cyclones, and the projections of sea level rise. Shoreline changes (erosion and accretion) were also addressed because they are associated with tropical cyclones and sea level rise. Our second aim was to evaluate the impact of human activities on the coasts. We thus assessed human encroachment (populations and settlements), the state of conservation of natural ecosystems, and the presence of coastal armoring used to protect the coasts from erosion and flooding. Our third goal was to identify advances and gaps in management and mitigation practices and propose better and more effective coastal management responses and strategies that avoid reaching conditions of no return. These extreme no-return conditions mean we can no longer recover the coasts’ structure and functionality, which would have unimaginable consequences for human settlements. Dealing with extreme conditions is particularly relevant, especially considering the millions of people living near or at the coast (and the many more who visit the coasts in their leisure time) worldwide and in Mexico.

## 2. Methods

### 2.1. Pressures: Environmental dynamics

#### 2.1.1. Tropical cyclones.

Information from the National Center for Disaster Prevention (CENAPRED in Spanish) was used to track hurricanes in Mexico (http://www.atlasnacionalderiesgos.gob.mx/descargas/?dir=). The historical record of tropical storms and hurricanes that have hit Mexico spans from 1949 to 2012 in the Atlantic basin and from 1950 to 2012 in the Pacific. We focused on hurricanes categories 1-5 for both basins because these are the most damaging tropical cyclones. We analyzed the yearly frequency of tropical cyclones per state from the publicly available data of the NOAA (National Oceanic and Atmospheric Administration) from 1863 to 2022 (https://www.coast.noaa.gov/hurricanes/#map=4/32/-80).

#### 2.1.2. Sea level rise projections.

Calculations were made for Mexico from the estimates reported by the IPCC [33] (https://www.ipcc.ch/report/ar6/syr/downloads/report/IPCC_AR6_SYR_LongerReport.pdf), using the RCP 8.5 scenario as a reference, which predicts a rise of 1.01 m by 2100 [33,34]. We developed a simple coastal flooding model (“bathtub model”) with the Digital Elevation Model of Mexico from the National Institute of Geography and Statistics (INEGI in Spanish) (https://www.inegi.org.mx/app/geo2/elevacionesmex/). This simple model does not consider other processes affecting coastal flooding, such as flow velocity, the geodynamics of the coast, or tides (that are microtidal for most of Mexican coastlines). Nevertheless, it helps identify areas exposed to flood risk [35–37].

#### 2.1.3. Shoreline changes.

Shoreline changes [38] determined long-term shoreline changes (1984–2016) on sandy beaches worldwide, using satellite images at a resolution of 500 m. To determine the shoreline change rate (m/yr), they applied linear regression to the shoreline positions of each transect. We used this data set to identify three trends in shoreline change rates on Mexican coasts: accretion (>0.5 m/yr), erosion (<-0.5 m/yr), and stable (-0.5 to 0.5 m/yr). In our analysis, we considered only change rates with uncertainties of less than 3 m/year, based on the values reported in the dataset.

### 2.2. Pressures: human impacts

#### 2.2.1. Coastal population and settlements.

We used the georeferenced population censuses of 1990, 2000, 2010, and 2020 from the INEGI database (https://www.inegi.org.mx/programas/ccpv/2020/). We focused on coastal states and their coastal populations, defined as all populations located on the coast at ≤ 10 m above sea level. The population growth rate (PGR) between two points in time, *t*_1_ and *t*_0_, was calculated for inland and coastal settlements using the following equation:


$$PGR=\left(\frac{P1-P0}{P0}\right)*100$$

where *P*_1_ and *P*_0_ are the number of inhabitants at times *t*_1_ and *t*_0_, respectively, and the time interval (*t*_1_‐*t*_0_) represents 10 years. Thus, it represents the percent change in ten years.

#### 2.2.2. Ecosystem condition.

From the information available regarding the state of conservation of coastal ecosystems in Mexico, we focused on mangroves and coastal dunes, ecosystems found on the terrestrial side of the coasts. The distribution and state of conservation of coastal dunes was obtained from [39], and the information for mangroves was gathered from the National Commission for the Knowledge and Use of Biodiversity (CONABIO in Spanish), published in 2020 (http://www.conabio.gob.mx/informacion/gis/).

#### 2.2.3. Coastal armoring.

We used photo interpretation and Geographic Information System methods for mapping defense structures and ports and later for data processing (GIS) (QGIS for Windows 10, V 2009). We performed all the estimations and analyses with the available georeferenced aerial photographs for 1995 and 2019. The 1995 images were satellite orthophotos (294), and the 2019 images were obtained from Google Earth Pro (Landsat/Copernicus satellites). The satellite images were georeferenced using the WGS 84 reference and the EPSG:4326 ellipsoid. The images were computer-rectified to eliminate scale and distortion effects [40,41]. The inventory considered different types of coastal protection structures (S1 1). The impact on coastal dynamics of each type is different [32], so they were inventoried separately. Docks’ external and internal perimeters were mapped for ports, minus floating structures, such as piers.

We created a database with each type of structure’s geographic location. We used MatLab to organize the information and determine the number of structures per state for each year. Finally, we calculated the rate of change in the number of structures per state from 1995 to 2019.

## 3. Results

### 3.1. Environmental dynamics

#### 3.1.1. Tropical cyclones.

**Fig 1:**
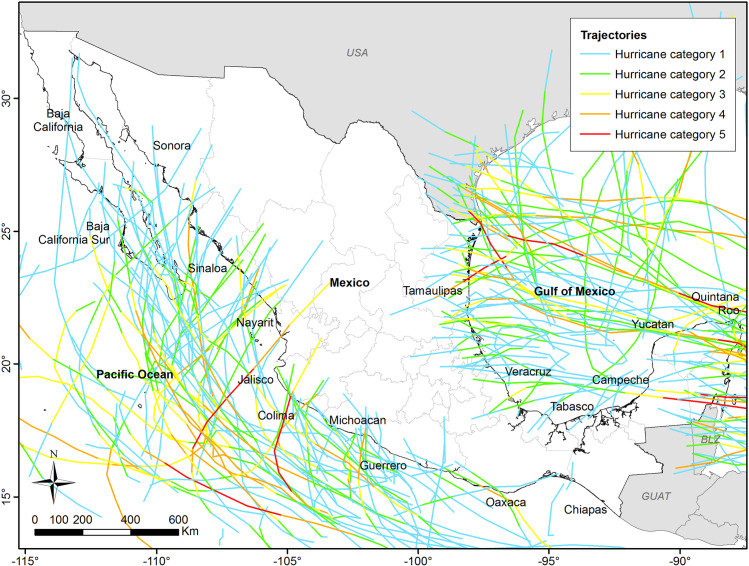
Trajectories of hurricanes making landfall on Mexican coasts 1949–2012. Data from CENAPRED, Mexico.

From 1863 to 2022, 386 tropical cyclones landed on Mexican coasts, 206 on the Pacific, and 180 on the Atlantic (Fig 1; Table 1). Most of the storms were tropical depressions and tropical storms, but hurricanes also occurred often (Table 1). Hurricanes of category 1 to 3 (gusts of wind ranging from 74 to 129 mph) were more frequent in the Pacific than in the Atlantic. However, intense hurricanes (categories 4 and 5 with winds ranging from 130-157 mph and higher) occurred more in the Atlantic than the Pacific (Fig 1, Table 1), especially hurricanes of Category 5 (Table 1). The state most frequently affected by hurricane Category 5 is primarily Quintana Roo, located on the Atlantic coast (Mexican Caribbean). In turn, tropical storms are widespread along many states, both along the Pacific (Baja California Sur, Oaxaca, and Sinaloa) and the Atlantic (Quintana Roo, Tamaulipas, and Veracruz) (Table 1).

**Table 1:** Number of tropical cyclones per state per category from 1863 to 2022.

		Category						
Pacific Ocean	State	TD	TS	H1	H2	H3	H4	H5
	Baja California	0	3	2	1	0	2	0
	Baja California Sur	2	15	14	8	8	12	0
	Chiapas	1	2	2	0	0	1	2
	Colima	0	3	2	0	1	1	0
	Guerrero	0	7	11	2	0	1	0
	Jalisco	0	4	6	2	2	0	1
	Michoacán	2	4	3	4	0	2	0
	Nayarit	0	0	0	0	1	1	1
	Oaxaca	6	12	2	5	0	1	0
	Sinaloa	1	12	10	7	3	6	2
	Sonora	1	1	1	0	0	0	0
	**Total**	**13**	**63**	**53**	**29**	**15**	**27**	**6**
Atlantic Ocean								
	Campeche	2	6	1	2	0	1	0
	Quintana Roo	2	24	13	12	13	8	7
	Tabasco	0	2	1	0	0	0	0
	Tamaulipas	7	17	8	4	2	0	3
	Veracruz	3	22	9	5	0	0	0
	Yucatán	0	3	2		1	0	0
	**Total**	**14**	**74**	**34**	**23**	**16**	**9**	**10**

TD =  tropical depression; TS =  tropical storm; H1 =  Hurricane category 1; H2 =  Hurricane category 2; H3 = Hurricane category 3; H4 =  Hurricane category 4 and H5 =  Hurricane category 5.

Data from NOA-National Hurricane Center (National Oceanic and Atmospheric Administration). Historical hurricane tracks, NOAA from Mexico. https://www.coast.noaa.gov/hurricanes/#map=4/32/-80.

#### 3.1.2. Sea level rise projections.

**Fig 2:**
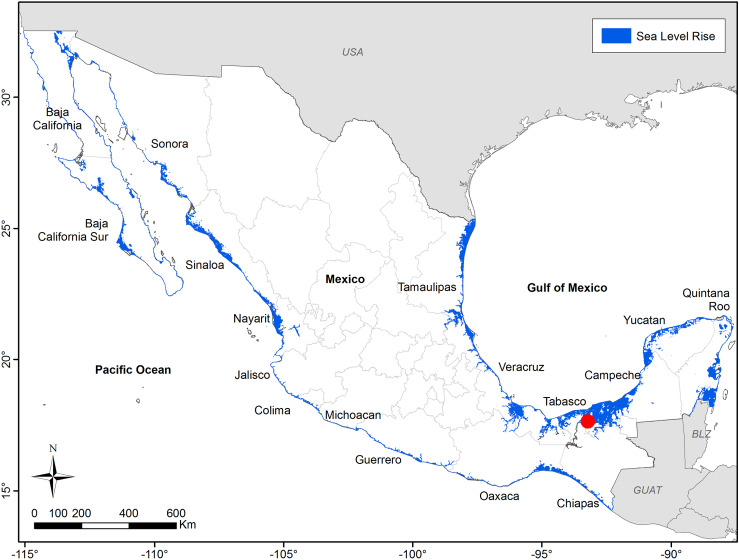
SLR projections for 2100 and RCP 8.5 scenario (estimated rise of 1.01 m). Data from IPCC (2023).

According to the 1.01 sea level increment predicted by the IPCC (2023), many Mexican coasts will be flooded by 2100. The Gulf of Mexico and the Caribbean are the coastlines most likely to be flooded by the sea, especially the southeastern Gulf of Mexico. This projection is of concern since many large settlements are here, notably Villahermosa, the capital of the state of Tabasco (Fig 2).

#### 3.1.3. Shoreline changes.

**Fig 3:**
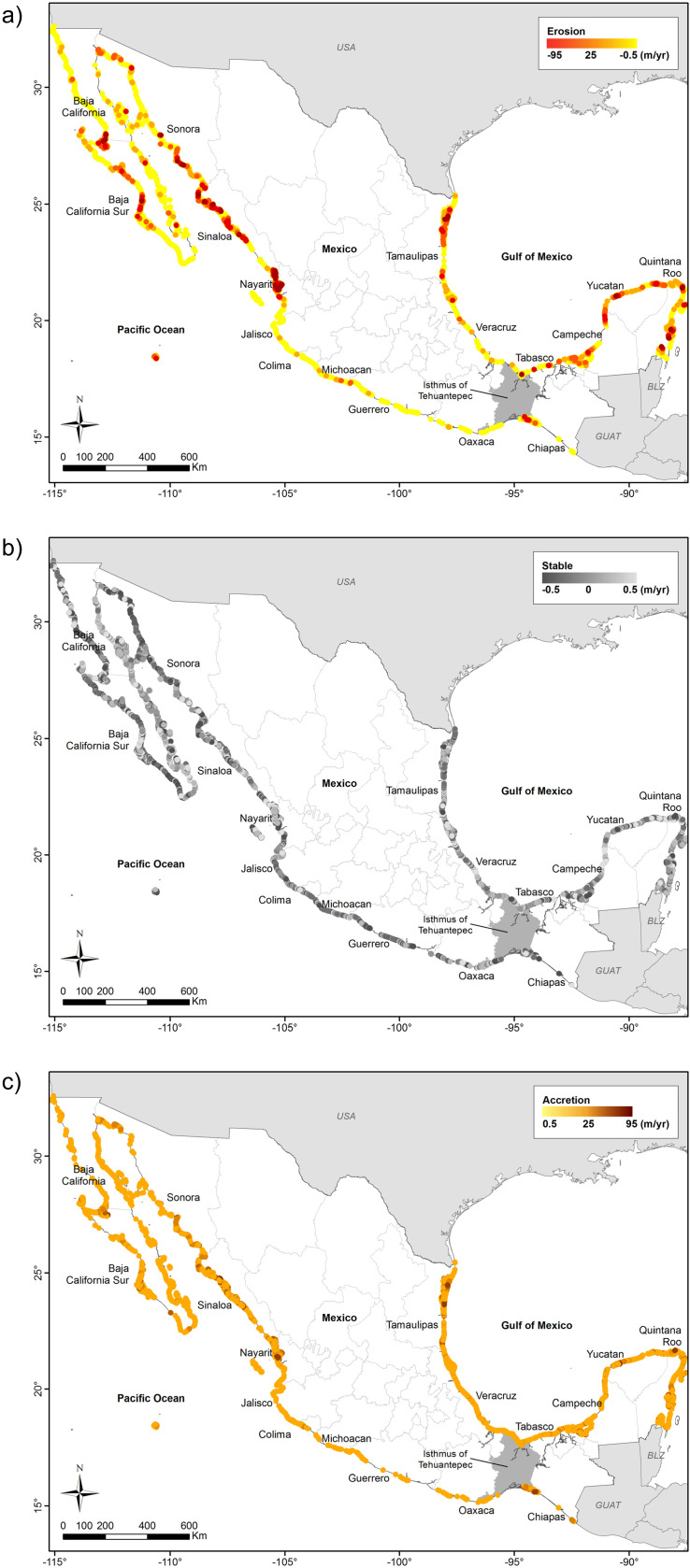
Shoreline change rates trends along the Mexican coast. (a) Erosion ( <-0.5 m/yr). (b) Stable (-0.5 to 0.5 m/yr). (c) Accretion (>0.5 m/yr).

Using the results of [38], Fig 3 shows the rates of shoreline change (a) accretion, (b) stable, and (c) erosion. The coasts of the southern Pacific are very stable, except around the Isthmus of Tehuantepec (between Oaxaca and Chiapas). The coasts of the northern Pacific have more dynamic trends, particularly in many regions of the states of Sinaloa and Nayarit, where more erosion was recorded compared with other states. Dynamic erosion patterns and accretion occur north of the Gulf of Mexico and the Yucatan Peninsula. At the same time, the coast of the central part of the Gulf of Mexico is more stable. Accreting coasts occur along the coasts of Nayarit, with some areas along other states such as Yucatán and Tamaulipas.

### 3.2. Human impacts

#### 3.2.1. Coastal population and settlements.

**Fig 4:**
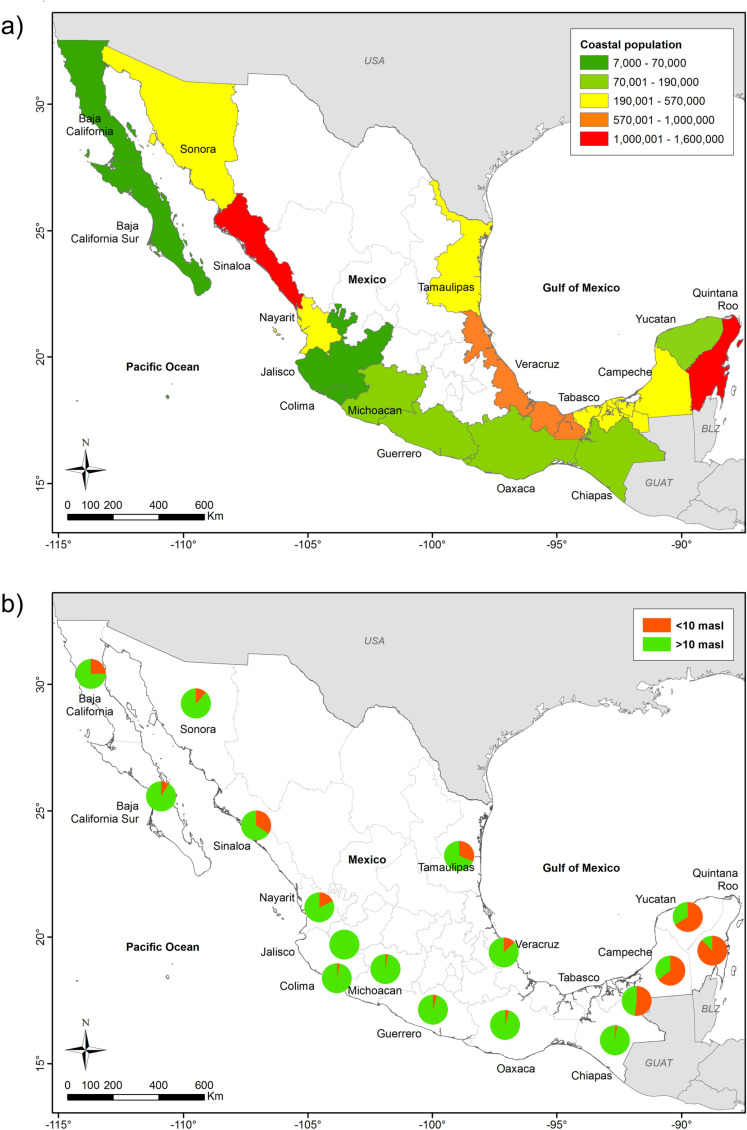
Population along the Mexican coasts. (a) Population found in LECZ (at ≤ 10 m above sea level). (b) Percent of population in LECZ (orange) compared to the total population of each state (green).

**Fig 5:**
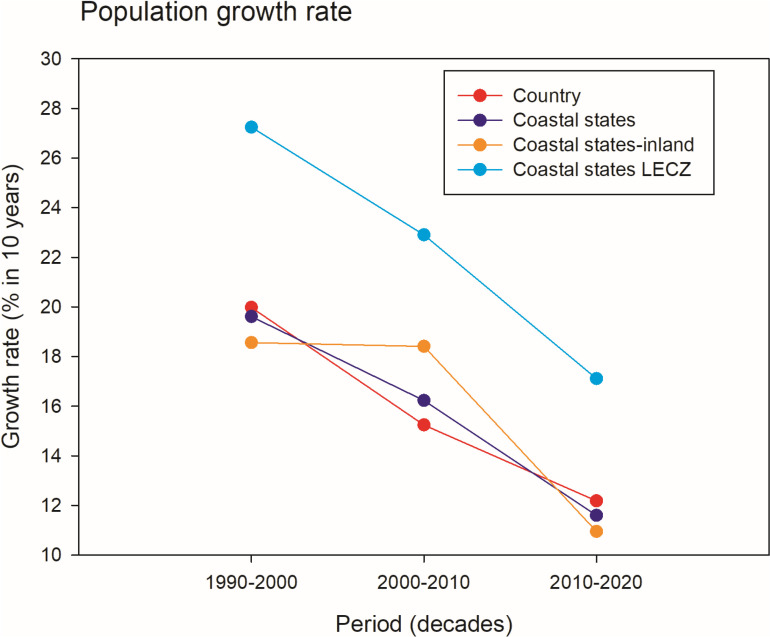
Population growth rates for Mexico, considering the country and the coastal states.

In Mexico, 6,399,268 people live in the low elevation coastal zones (LECZ) (defined as <  10 m above sea level and 20 km inland from the shoreline), which is 6% of the population living in 10.3% of the national surface area. Fig 4 shows that some coastal states are more densely populated than others: Sinaloa on the Pacific and Quintana Roo on the Caribbean have the most densely populated coasts, followed by Veracruz on the Gulf of Mexico. Baja California, Baja California Sur, Colima, and Jalisco, all in the Pacific basin, have the lowest population densities. The proportion of inhabitants living in LECZ compared to the total population per state is very high in the Yucatan Peninsula (especially Quintana Roo), which has the most popular tourist resorts (Cancun and the Mayan Riviera). The population growth rate has fallen over recent decades but has remained highest in the LECZ (Fig 5).

#### 3.2.2. Ecosystem condition.

**Fig 6:**
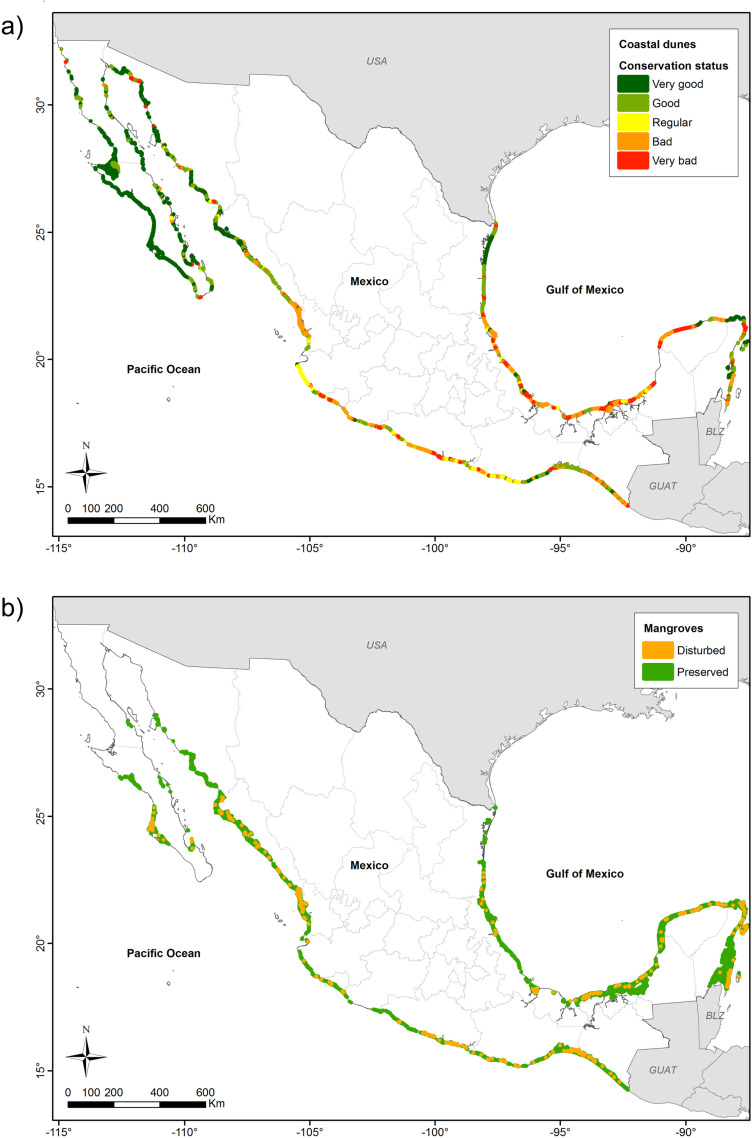
Distribution and state of conservation of the most abundant terrestrial coastal ecosystems in Mexico. (a) Coastal dunes. (b) Mangroves.

The distribution of mangroves and coastal dunes is broad along Mexico’s coasts, covering almost 800,000 hectares each. The state of conservation of these two ecosystems is very different, mainly because mangroves are legally protected, while coastal dunes are not. The coastal dunes in the north of Mexico are in good condition overall, but not so those on the south of the Pacific, Gulf of Mexico, and the Caribbean (Fig 6a), which are in a bad or very bad condition by [39]. Mangroves, in contrast, seem to be in a better-preserved condition throughout Mexico, although some areas in southeastern Mexico and the South Pacific are disturbed (Fig 6b).

#### 3.2.3. Coastal armoring.

The distribution of coastal armoring along the Mexican coasts is uneven (S2). The Yucatán Peninsula has the most coastal protection infrastructure (55% of all the coastal infrastructures in Mexico), especially the states of Yucatán (35%) and Quintana Roo (14%), although Veracruz, on the Gulf of Mexico, also has many. On the Pacific, Sonora and Baja California Sur also have quite a high number of structures. The increase in the number of these is very high in Baja California, Sonora, Jalisco, Yucatán, Campeche, and Quintana Roo and very low elsewhere.

The types and abundance of coastal structures inventoried vary by state (Table 2). For instance, Veracruz and Sonora have the most jetties, whereas most groins are in Yucatán. Baja California Sur and Sinaloa have the largest number of seawalls, and Baja California and Sonora have the most ports. Breakwaters are common in Baja California Sur, Jalisco, and Quintana Roo.

**Table 2:** Presence of different coastal structures along the coasts of Mexico in 1995 and 2019.

Coast	State	Jetty		Groin		Quay		Seawall		Port		Breakwater		Total	Total
		1995	2019	1995	2019	1995	2019	1995	2019	1995	2019	1995	2019	1995	2019
Pacific Coast	Baja California	3	5	1	6	3	7	2	2	4	6		2	13	28
	Baja California Sur	8	12	17	**28**	8	13	2	7	7	10	5	7	47	77
	Chiapas				1					1	1			1	2
	Colima	3	5	1	1	1	1			2	2			7	9
	Guerrero			2	2	19	**27**	3	3	2	3		1	26	36
	Jalisco	1	1	10	**26**	4	5			2	2		5	17	39
	Michoacán			3	3					1	1			4	4
	Nayarit	7	8	1	7		1			3	3		1	11	20
	Oaxaca	8	8	4	5	2	3			2	2		1	16	19
	Sinaloa	7	11	10	17		2	3	4	3	4		2	23	40
	Sonora	3	**29**	4	19	11	17	3	3	9	12			30	80
	Total	40	79	53	115	48	76	13	19	36	46	5	19	195	354
Atlantic coast	Campeche	5	11	24	27	10	17		1	2	3	1	3	42	62
	Quintana Roo	7	10	21	47	53	**74**		1	5	9	2	5	88	146
	Tabasco	2	4							1	1			3	5
	Tamaulipas	14	16							1	1			15	17
	Veracruz	**30**	**37**	**37**	**43**				2	3	3	1	2	71	87
	Yucatán	12	12	**124**	**322**	16	**22**			3	3			155	359
	Total	70	90	206	439	79	113	0	4	15	20	4	10	374	676
Country total		110	169	259	554	127	189	13	23	51	66	9	29	569	1,030

Bold type shows the highest values per structure.

## 4. Discussion

This is the first study performed at a national level to analyze with a multidisciplinary approach the different pressures facing Mexican coasts and the conservation status of some of its coastal ecosystems. It helped us find advances and gaps in the current condition and management practices. Our findings show that the meteorological conditions on Mexican coasts are worsening: tropical cyclones occur more frequently and with greater intensity, and sea level rise projections suggest several vulnerable areas. Furthermore, shoreline erosion over recent decades is also a matter of concern in many places.

In addition to the above, as the coastal population grows, the impact of human activities associated with maritime commerce, increasing sun, sand, and sea tourism, and growing coastal armoring make the degradation of coastal ecosystems more challenging. Ecosystem degradation exacerbates societal vulnerability because of decreased storm protection. The increased exposure (more humans at the coast) and vulnerability (reduced protection from ecosystems owing to their loss and degradation, and human induced erosion) result in more significant risks for coastal populations in Mexico [42]. These factors show that the call for action for better management of the Mexican coasts is urgent.

Decision-makers and society must recognize that combining socioeconomic needs with ecological processes is vital because coastal biodiversity and ecosystem services are irreplaceable. However, they are only sometimes recognized as necessary for society even though many human interests are affected by the degradation of the coastal ecosystems. To tackle the enormous range of threats facing human settlements on the coast and the natural ecosystems there, we need transdisciplinary research and radical management actions now and in the immediate future. The challenges vary geographically, as summarized in S3; thus, the actions required are site-specific.

The information in this study enables us to identify critical directions for future research and pinpoint actions required to improve the condition of Mexican coasts. Better coastal management should thus reduce the risk of flooding and erosion for human settlements and promote the conservation of coastal ecosystems. We have grouped these strategies into two sets of research directions and actions that would help humans live with nature and make the coasts more sustainable.

### 4.1. Living with nature

#### 4.1.1. Dynamic coasts.

As coasts are naturally dynamic, coastal ecosystems are frequently exposed to various disturbance events, such as storms, waves, winds, tides and erosion. However, the ecosystems will regenerate, rebuild, and expand if the natural flow of water, sediments, nutrients, and propagules is free to occur [16]. Humans need to be aware of these dynamics and live with them instead of trying unsuccessfully to control and avoid them.

The environmental dynamics of the coastal states differ throughout Mexico. For instance, Baja California Sur and Quintana Roo are frequently affected by tropical cyclones. Regarding the projections of sea level rise, it is likely to affect the Gulf of Mexico and the Caribbean coasts. However, erosion is critical in Sinaloa, Nayarit, Yucatán, and Quintana Roo. These states require new paradigms of coastal management more urgently than others to progressively incorporate the natural dynamics of the coasts (biotic and abiotic aspects), ideally based on sound ecological science. Better capacities in diagnosis and predictions are necessary to understand the ecological consequences of human activities and disturbances on natural ecosystems and their dynamics.

#### 4.1.2. Coastal protection.

The accelerated human encroachment on the coasts and increasing flooding and erosion risks associated with extreme hydrometeorological events means the coasts’ protection is becoming critical worldwide. However, there is no consensus on the meaning of “coastal protection” [43]. To some, it may mean building structures to halt coastal erosion and protect properties and human assets. However, to others, it may mean allowing coastal ecosystems to function naturally and conserving their dynamics while infrastructure and population are translated landwards [44].

The outcomes of each of these alternatives are contrasting. A hard-protected coastline will have modified sediment and hydrodynamic flows, and erosion problems may often move down drift [45]. In turn, if natural processes shape the coasts, these can respond to environmental fluctuations as long as their dynamic nature can occur. For these reasons, creating and restoring natural coastal ecosystems is considered a cost-effective means of protection, which is sustainable and ecologically sound [4]. Additional ecosystem services include scenic beauty, recreation, water quality, and habitat [46,47], a further advantage. Hence, maintaining balanced natural connectivity in mass and energy fluxes must be recognized as one of the main attributes of any coastal protection project [9].

Society can adopt many strategies for decreasing flooding and erosion risks [48]. These should aim at reducing the risks, increasing resilience (the ability of a system to continue operating and return to a pre-disturbance condition), and reducing the vulnerability of a system. In this sense [48], propose three categories of strategies for coastal protection: Protect, Accommodate, and Retreat. *Protection* includes coastal armoring, soft engineering solutions (such as beach nourishment), and ecosystem-based solutions (restoring and protecting coastal ecosystems). *Accommodation* strategies refer to land regulation systems and preparedness programs. The *Retreat* option means the realignment of infrastructure to allow the natural dynamics of the coast to occur. Protection actions become most relevant for coastal settlements and infrastructure, whereas accommodation and retreating refer to land use and development policies. We will focus on the protection option as this is the most urgently needed, given the risks to which human settlements are already exposed.

The protection offered by green infrastructure can be an effective solution, at least in the less extreme but more frequent circumstances. [9] proposed that such protection varies according to the degree of naturalness of the solution: Nature Reclamation, Engineered Ecosystems, Ecologically Enhanced Engineering, and De-engineering/Relocation. Nature Reclamation (NR) is the ideal solution when habitat conservation and restoration are feasible. Where and when NR is not possible, Engineered Ecosystems, which are rehabilitated ecosystems, help recover critical services without reaching the level of complexity of natural systems. Ecologically Enhanced Engineering is functional where space and risk conditions necessitate traditional hard and/or soft engineering measures. These are modified to change physical processes, indirectly producing certain benefits from natural processes that are maintained or adapted to imitate natural ecosystems. De-engineering/Relocation: It is sometimes more convenient to relocate human interests to more convenient locations and conditions, restoring the system and moving towards a more natural functioning. These alternatives can be combined (hybrid solutions), depending on the specific conditions of the site to be protected and taking into consideration the space and time required to implement them, the related costs, and the allowable uncertainties in the response of the local economy and the coastal environment [9].

From the data analyzed (1995 and 2019), it is evident that coastal armoring in Mexico has increased, most noticeably on the coasts of Yucatán, Quintana Roo, Veracruz, Sonora, Baja California Sur, and Campeche. However, questions arise on the necessity, quality, and consequences of such prolific coastal armoring; a diagnosis of the performance of this coastal armoring is needed, and if it is environmentally and socially adequate [32]. Future projects should explore new, softer coastal defense approaches as a more proactive means of protecting the coasts from erosion and flooding.

Whatever type of coastal protection is selected, monitoring its effectiveness and environmental impacts is essential to avoid unwanted side effects, such as chronic downdrift erosion or biodiversity loss. We should not repeat past wrongful protection schemes. Instead, we need to rely on recent knowledge, and the best management and protection practices should be selected based on local conditions and scientific evidence [10].

#### 4.1.3. Restore and preserve coastal ecosystems.

The degradation and loss of natural ecosystems lead to biodiversity loss and reduced ecosystem functioning, resulting in a decrease in the potential of the ecosystems to buffer and protect the coast from the impact of meteorological disturbances. Additionally, the scenic beauty, fisheries, and water filtration may also be negatively affected, probably leading to socioeconomic costs [16,46,49]. Restoring coastal ecosystems would provide many ecosystem services and goods (storm protection, recreation, nurseries, and water quality) worth millions of dollars [50]. A further benefit of protecting dunes is their importance in the biodiversity of Mexico’s flora. For instance, Mexico’s coastal dunes cover approximately 800,000 ha, representing 0.04% of the nation’s total land surface [39]. Nevertheless, because of their topographic heterogeneity, variability in weather regimes, and diversity of vegetation types, they host 2072 plant species [51], the equivalent of 9.4% of the Mexican flora.

The latest advances in science and technology are vital to protect and restore Mexico’s coasts and their natural ecosystems from further degradation and loss. Critical issues in ecosystem protection policies include the creation of biological corridors to promote genetic exchange, the restoration of fragments and more extensive areas with natural ecosystems, controlling the arrival and expansion of invasive species, and protecting endangered or threatened species [48]. Current research shows that emphasis on protecting coastal dunes and mangroves in the southeastern states of Mexico is vital because degradation and losses are most severe.

### 4.2. Making the coasts a sustainable place to live

#### 4.2.1. Coastal cities in a dynamic setting.

Many coastal cities are exposed to erosion and flooding by storm surges and sea level rise and are thus very vulnerable. Sustainable coastal cities need to thrive and function according to the natural dynamics of their environment, which are impossible to control. So, the society (inhabitants, visitors, and authorities) must be prepared for a dynamic and unpredictable environment [10].

As the risk of flooding and erosion in coastal cities increases, vulnerability assessment and adaptation options are urgently needed [52,53]. For example, we can avoid new constructions in areas prone to flooding or erosion. Alternatively [50], recommend replacing these low-lying areas with natural ecosystems (such as wetlands) that trap sediments to rebuild the land or insisting on new buildings standing on piles to survive moderate flooding. On the other hand, new tourist destinations should be planned better at the national level, considering how best to combine the preservation of natural coastal ecosystems with the region’s socioeconomic development while maintaining a low-risk condition. The construction of entirely new sustainable cities designed explicitly for tourism is a once-in-a-lifetime opportunity to have cities adapted to coastal dynamics and natural hazards and which, additionally, could have high-performance green buildings (using renewable energy, for example) and adequate transportation with zero or reduced CO_2_ emissions [16]. Combining infrastructure with the buffering service natural ecosystems provide will help coastal cities become more sustainable and less vulnerable to natural hazards.

#### 4.2.2. Rebuild the social capital.

[48] analyzed community perception and community adaptation to climate change in coastal areas of Mexico. They encountered difficulties in effective adaptation measures: institutional discrepancies regarding conservation and development, weak governance structures that impede having an informed society, and the overexploitation of natural resources. A successful coastal protection project should consider using local resources and the participation of local actors [9]. Better organization and planning are needed to achieve a sustainable coast. Additionally, communities in sustainable cities should be fair, respectful, and tolerant.

#### 4.2.3. Dealing with risk.

Finally, the inhabitants and authorities of coastal cities should not be misled: a zero-risk coast does not exist and never will. Consequently, coastal cities should be prepared for the uncertainties derived from the dynamic and unpredictable setting [10].

An unfortunate and recent example occurred while we were writing this article. On October 24^th^, 2023, category 5 hurricane Otis landed directly on Acapulco’s significant touristic coastal city (on the Mexican Pacific), with 800,000 inhabitants and 50% tourist occupancy. Otis behaved unexpectedly: within a few hours, it developed from being a tropical storm to a hurricane category 5, possibly because of abnormally high temperatures on the surface of the Pacific Ocean. It is among the most damaging hurricanes ever affecting Mexico; the first hurricane category 5 that has ever landed on Acapulco in the last hundred years. Indeed, no natural ecosystem could have avoided the dreadful damages caused by 350 km/hr gusts of winds. However, perhaps, as Acapulco is rebuilt (hopefully very soon), we can think of better ways of reconstructing the destroyed buildings by improving construction norms and window frames (most of them destroyed) and restoring degraded ecosystems so that this iconic coastal city is even more beautiful than before Otis. Acapulco is an unfortunate example of why, now, more than ever, coastal cities must be prepared for the unexpected.

## 5. Conclusions

Processes such as the availability of sediment in the swash zone, wind, waves, tides, and storm surges can significantly influence shoreline changes. However, along the Mexican coasts, the escalating threats and pressures discussed here are primarily associated with more extreme environmental dynamics, such as tropical cyclones and sea level rise. These factors, combined with intensified human activities—such as encroachment, ecosystem loss and degradation, and coastal armoring—further exacerbate the situation. The consequences are diverse, including loss of biodiversity and ecosystem services, increased vulnerability, and risk. Indeed, the voracious growth of human activities, especially tourism on the coasts, is seriously affecting many coastal ecosystems, resulting in either degradation or loss. Such changes could have irreversible consequences for ecosystem functions, including those considered most necessary for society: scenic beauty, storm protection, water filtration, and providing habitats for fisheries.

One alternative to address the challenges in this study is the concept of “living with nature,” which means that humans must learn to live on naturally dynamic coasts while restoring and protecting natural coastal ecosystems effectively, which will help provide nature-based storm protection. Furthermore, we need to make the coasts a sustainable place to live, which means adapting cities to a dynamic environment, rebuilding the social capital, and learning to live with risk. The pressures and extreme conditions the Mexican coastlines face vary geographically; thus, the challenges and needs for action are site-specific.

The socio-economic conditions of Mexico are similar to those of other developing, megadiverse countries, where primary activities (e.g., agriculture and extractive industries) and services (e.g., trade and tourism) are fundamental to the economy. In such countries integrated monitoring programmes of the seas and coasts are generally very limited. We believe that this study could be adapted, extended and applied in other countries that need to establish their own coastal management programmes at a national level.

## Supporting information

S1 date (CSV)

## References

[1] Murray NJ, Phinn SR, DeWitt M, Ferrari R, Johnston R, Lyons MB, et al. The global distribution and trajectory of tidal flats. Nature. 2019;565(7738):222–5. doi: 10.1038/s41586-018-0805-8 30568300

[2] Vousdoukas MI, Ranasinghe R, Mentaschi L, Plomaritis TA, Athanasiou P, Luijendijk A, et al. Sandy coastlines under threat of erosion. Nat Clim Chang. 2020;10(3):260–3. doi: 10.1038/s41558-020-0697-0

[3] Neumann B, Vafeidis AT, Zimmermann J, Nicholls RJ. Future coastal population growth and exposure to sea-level rise and coastal flooding—a global assessment. PLoS One. 2015;10(3):e0118571. doi: 10.1371/journal.pone.0118571 25760037 PMC4367969

[4] Temmerman S, Meire P, Bouma TJ, Herman PMJ, Ysebaert T, De Vriend HJ. Ecosystem-based coastal defence in the face of global change. Nature. 2013;504(7478):79–83. doi: 10.1038/nature12859 24305151

[5] Janssen S, Vreugdenhil H, Hermans L, Slinger J. On the nature based flood defence dilemma and its resolution: a game theory based analysis. Sci Total Environ. 2020;705:135359. doi: 10.1016/j.scitotenv.2019.135359 31838412

[6] James RK, Silva R, van Tussenbroek BI, Escudero-Castillo M, Mariño-Tapia I, Dijkstra HA, et al. Maintaining tropical beaches with seagrass and algae: a promising alternative to engineering solutions. BioScience. 2019;69(2):136–42. doi: 10.1093/biosci/biy154

[7] Martínez ML, Silva R, López-Portillo J, Feagin RA, Martínez E. Coastal ecosystems as an ecological membrane. J Coastal Res. 2020;95(sp1):97. doi: 10.2112/si95-019.1

[8] Silva R, Martínez ML, van Tussenbroek BI, Guzmán-Rodríguez LO, Mendoza E, López-Portillo J. A framework to manage coastal squeeze. Sustainability. 2020;12(24):10610. doi: 10.3390/su122410610

[9] Chávez V, Lithgow D, Losada M, Silva-Casarin R. Coastal green infrastructure to mitigate coastal squeeze. J Infrastruct Preserv Resil. 2021;2(1). doi: 10.1186/s43065-021-00026-1

[10] Silva R, Oumeraci H, Martínez ML, Chávez V, Lithgow D, van Tussenbroek BI, et al. Ten commandments for sustainable, safe, and w/healthy sandy coasts facing global change. Front Mar Sci. 2021;8. doi: 10.3389/fmars.2021.616321

[11] Zhu Z, Vuik V, Visser PJ, Soens T, van Wesenbeeck B, van de Koppel J, et al. Historic storms and the hidden value of coastal wetlands for nature-based flood defence. Nat Sustain. 2020;3(10):853–62. doi: 10.1038/s41893-020-0556-z

[12] Silva R, Martínez M, Hesp P, Catalan P, Osorio A, Martell R, et al. Present and future challenges of coastal erosion in Latin America. J Coastal Res. 2014;71(71):1–16.

[13] Agency C. The CIA World Factbook 2020–2021. Simon and Schuster; 2020.

[14] INEGI. Datos básicos de la geografía de Mexico. Instituto Nacional de Estadística, Geografía e Informática Mexico. 1991. Available from: http://internet.contenidos.inegi.org.mx/contenidos/productos/prod_serv/contenidos/espanol/bvinegi/productos/historicos/2104/702825221218/702825221218_1.pdf

[15] McLachlan A, Brown AC. The ecology of sandy shores. Burlington, MA: Academic Press; 2006.

[16] Martinez ML, Costanza R, Pérez-Maqueo OM, Silva R, Maximiliano-Cordova C, Chávez V, et al. Storm protection as a service from estuarine and coastal ecosystems. In: Baird D, Elliott M, eds. Treatise on Estuarine and Coastal Science (Second Edition), vol. 7. 2024:79–110. doi: 10.1016/b978-0-323-90798-9.00063-9

[17] Mendoza-González G, Martínez ML, Lithgow D, Pérez-Maqueo O, Simonin P. Land use change and its effects on the value of ecosystem services along the coast of the Gulf of Mexico. Ecol Econ. 2012;82:23–32. doi: 10.1016/j.ecolecon.2012.07.018

[18] Sytnik O, Stecchi F. Disappearing coastal dunes: tourism development and future challenges, a case-study from Ravenna, Italy. J Coast Conserv. 2014;19(5):715–27. doi: 10.1007/s11852-014-0353-9

[19] Kotwicki L, Weslawski J, Szaltynis A, Stasiak A, Kupiec A. Fine organic particles in a sandy beach system (Puck Bay, Baltic Sea). Oceanologia. 2005;47(2).

[20] Perry CT, Kench PS, Smithers SG, Riegl B, Yamano H, O’Leary MJ. Implications of reef ecosystem change for the stability and maintenance of coral reef islands. Global Change Biology. 2011;17(12):3679–96. doi: 10.1111/j.1365-2486.2011.02523.x

[21] Jänes H, Macreadie PI, Zu Ermgassen PSE, Gair JR, Treby S, Reeves S, et al. Quantifying fisheries enhancement from coastal vegetated ecosystems. Ecosyst Serv. 2020;43:101105. doi: 10.1016/j.ecoser.2020.101105

[22] Martínez ML, Vázquez G, Pérez-Maqueo O, Silva R, Moreno-Casasola P, Mendoza-González G, et al. A systemic view of potential environmental impacts of ocean energy production. Renew Sustain Energy Rev. 2021;149:111332. doi: 10.1016/j.rser.2021.111332

[23] Eckstein D, Künzel V, Schäfer L. The Global Climate Risk Index 2021. Bonn: Germanwatch. 2021.

[24] Costanza R, Anderson SJ, Sutton P, Mulder K, Mulder O, Kubiszewski I, et al. The global value of coastal wetlands for storm protection. Global Environ Change. 2021;70:102328. doi: 10.1016/j.gloenvcha.2021.102328

[25] INEGI. Censos y conteos de población y vivienda 2020. 2020.

[26] Lithgow D, Martínez ML, Gallego-Fernández JB, Silva R, Ramírez-Vargas DL. Exploring the co-occurrence between coastal squeeze and coastal tourism in a changing climate and its consequences. Tour Manage. 2019;74:43–54. doi: 10.1016/j.tourman.2019.02.005

[27] Maximiliano-Cordova C, Martínez ML, Silva R, Hesp PA, Guevara R, Landgrave R. Assessing the impact of a winter storm on the beach and dune systems and erosion mitigation by plants. Front Mar Sci. 2021;8. doi: 10.3389/fmars.2021.734036

[28] Schlacher TA, Dugan J, Schoeman DS, Lastra M, Jones A, Scapini F, et al. Sandy beaches at the brink. Divers Distrib. 2007;13(5):556–60. doi: 10.1111/j.1472-4642.2007.00363.x

[29] McGranahan G, Balk D, Anderson B. The rising tide: assessing the risks of climate change and human settlements in low elevation coastal zones. Environ Urban. 2007;19(1):17–37. doi: 10.1177/0956247807076960

[30] Salgado K, Martínez ML, Álvarez-Molina LL, Hesp P, Equihua M, Mariño-Tapia I. Impact of urbanization and landscape changes on the vegetation of coastal dunes along the Gulf of Mexico. Écoscience. 2021;29(2):103–16. doi: 10.1080/11956860.2021.1934299

[31] Luisa Martínez Ma, Mendoza-González G, Silva-Casarín R, Mendoza-Baldwin E. Land use changes and sea level rise may induce a “coastal squeeze” on the coasts of Veracruz, Mexico. Global Environ Change. 2014;29:180–8. doi: 10.1016/j.gloenvcha.2014.09.009

[32] Manno G, Anfuso G, Messina E, Williams AT, Suffo M, Liguori V. Decadal evolution of coastline armouring along the Mediterranean Andalusia littoral (South of Spain). Ocean Coast Manage. 2016;124:84–99. doi: 10.1016/j.ocecoaman.2016.02.007

[33] IPCC. Sections. In: Core Writing Team, Lee H, Romero J, editors. IPCC, 2023: Climate Change 2023: Synthesis Report. Contribution of Working Groups I, II and III to the Sixth Assessment Report of the Intergovernmental Panel on Climate Change. Geneva, Switzerland: IPCC. 2023. doi: 10.59327/ipcc/ar6-9789291691647

[34] Pachauri R, Allen M, Barros V, Broome J, Cramer W, Christ R, et al. Climate change 2014: synthesis report. Contribution of Working Groups I, II and III to the fifth assessment report of the Intergovernmental Panel on Climate Change. IPCC; 2014.

[35] Hansen J. The use of modelling tools to assess local scale inundation and erosion risk. CoastAdapt. National Climate Change Adaptation Research Facility, Gold Coast; 2016.

[36] Kulp S, Strauss BH. Rapid escalation of coastal flood exposure in US municipalities from sea level rise. Clim Change. 2017;142(3–4):477–89. doi: 10.1007/s10584-017-1963-7

[37] NOAA, Sea level rise viewer. 2023. Available from: https://coast.noaa.gov/slr/.

[38] Luijendijk A, Hagenaars G, Ranasinghe R, Baart F, Donchyts G, Aarninkhof S. The State of the World’s Beaches. Sci Rep. 2018;8(1):6641. doi: 10.1038/s41598-018-24630-6 29703960 PMC5923213

[39] Martínez M. Diagnóstico de las dunas costeras de México. Comisión Nacional Forestal; 2014:390.

[40] Crowell M, Leatherman S, Buckley M. Shoreline change rate analysis: long term versus short term data. Shore Beach. 1993;61(2):13–20.

[41] Moore LJ. Shoreline mapping techniques. J Coast Res. 2000:111–24.

[42] Salgado K, Martínez ML, Pérez-Maqueo O, Equihua M, Mariño-Tapia I, Hesp P. Estimating storm-related coastal risk in Mexico using Bayesian networks and the occurrence of natural ecosystems. Nat Hazards. 2024;120(6):5919–40. doi: 10.1007/s11069-024-06460-0

[43] Cooper JAG, Mckenna J. Working with natural processes: the challenge for coastal protection strategies. Geograph J. 2008;174(4):315–31. doi: 10.1111/j.1475-4959.2008.00302.x

[44] Phillips MR, Jones AL. Erosion and tourism infrastructure in the coastal zone: problems, consequences and management. Tour Manage. 2006;27(3):517–24. doi: 10.1016/j.tourman.2005.10.019

[45] Schoonees T, Gijón Mancheño A, Scheres B, Bouma TJ, Silva R, Schlurmann T, et al. Hard structures for coastal protection, towards greener designs. Estuaries Coasts. 2019;42(7):1709–29. doi: 10.1007/s12237-019-00551-z

[46] Everard M, Jones L, Watts B. Have we neglected the societal importance of sand dunes? An ecosystem services perspective. Aquat Conserv: Mar Freshw Ecosyst. 2010;20(4):476–87. doi: 10.1002/aqc.1114

[47] Salgado K, Martinez ML. Is ecosystem-based coastal defense a realistic alternative? Exploring the evidence. J Coast Conserv. 2017;21(6):837–48. doi: 10.1007/s11852-017-0545-1

[48] Escudero M, Mendoza E. Community perception and adaptation to climate change in coastal areas of Mexico. Water. 2021;13(18):2483. doi: 10.3390/w13182483

[49] Barbier EB, Hacker SD, Kennedy C, Koch EW, Stier AC, Silliman BR. The value of estuarine and coastal ecosystem services. Ecol Monogr. 2011;81(2):169–93. doi: 10.1890/10-1510.1

[50] Costanza R, Mitsch WJ, Day JW Jr. A new vision for New Orleans and the Mississippi delta: applying ecological economics and ecological engineering. Front Ecol Environ. 2006;4(9):465–72. doi: 10.1890/1540-9295(2006)4[465:anvfno]2.0.co;2

[51] Espejel I, Jiménez-Orocio O, Castillo-Campos G, P. Garcillán P, Álvarez L, Castillo-Argüero S, et al. Flora en playas y dunas costeras de México. Acta Bot Mex. 2017;(121):39–81. doi: 10.21829/abm121.2017.1290

[52] Hallegatte S, Green C, Nicholls RJ, Corfee-Morlot J. Future flood losses in major coastal cities. Nat Clim Change. 2013;3(9):802–6. doi: 10.1038/nclimate1979

[53] Le TDN. Climate change adaptation in coastal cities of developing countries: characterizing types of vulnerability and adaptation options. Mitig Adapt Strateg Glob Change. 2019;25(5):739–61. doi: 10.1007/s11027-019-09888-z

